# Effect of Wheat Replacement by Pulse Flours on the Texture, Color, and Sensorial Characteristics of Crackers: Flash Profile Analysis

**DOI:** 10.1155/2022/2354045

**Published:** 2022-08-18

**Authors:** Danai Ioanna Koukoumaki, Konstantinos Giannoutsos, Putu Virgina Partha Devanthi, Panagiotis Karmiris, Sophia Bourni, Anastasia Monemvasioti, Vassiliki Psimouli, Dimitris Sarris, Konstantinos Gkatzionis

**Affiliations:** ^1^Laboratory of Consumer and Sensory Perception of Food & Drinks, Department of Food Science and Nutrition, School of the Environment, University of the Aegean, Metropolite Ioakeim 2, GR 81400 Myrina, Lemnos, Greece; ^2^Indonesia International Institute for Life Sciences, Jakarta 13210, Indonesia

## Abstract

Pulse flours are growing in popularity as alternatives to wheat in bakery products due to their high protein and nutritional value. However, the effect of different pulse species and substitution on sensory perception is unclear. The sensory perception of crackers made by partially replacing wheat with chickpea (40-80%) and lupin flour (10-30%) was evaluated using Flash profile analysis in association with instrumental analysis of texture and color. Flash profile analysis was conducted in Greece and Indonesia in order to allow culture comparison of the profiling of the samples and language by the subjects of the panel. Lightness (L∗) and hardness of crackers were decreased by the addition of pulses. Flash profile analysis indicated an association among color, texture, and sensory perception by judges. Derived attributes were associated with the physicochemical characteristics and raw materials of crackers for both panels. GPA analysis of Greek panel indicated that increasing the replacement of wheat led to the generation of more attributes regardless of pulse species, while the Indonesian panel was able to detect differences among pulse species.

## 1. Introduction

In recent years, there has been a tendency towards the substitution of wheat and formulation of products using pulses (flours of grain legumes) [1]. Numerous pasta and bakery products, previously based on wheat flour nowadays partly contain or are made exclusively from pulses [2]. Pulses such as chickpeas, peas, soy, lupin, lentil, and beans have been researched as ingredients in bakery [2–10] in order to create products that claim nutritional benefits.

Among bakery products, crackers are high in demand snack foods suitable for the inclusion of pulses [11, 12]. Numerous studies have examined the addition of different kinds of pulses [13, 14], different ratios of pulses [15], and different mixtures of pulses with other materials [16] in physicochemical properties and overall acceptability in bakery snacks. Thus, research is necessary on the consumer perception of pulse-based crackers in comparison to traditional wheat-based recipes. For example, when composite flours were used in biscuits, the increased hardness as shown by texture analysis resulted in higher acceptability, probably due to being perceived as crunchier [17]. In a study of high plant protein snacks, the increase of added peas resulted in an increase of crispiness [18]. When pulse flours were used in extruded snacks, terms such as “hard” and “crumbly” were dominant in describing samples made with chickpea and green pea flour [19]. Overall, it appears that consumers are eager to try and evaluate positively innovative bakery products with flours alternative to wheat [20, 21]. However, sensory evaluation is influenced by different factors such as whether or not there is information about the samples prior to assessment by the panel [22]. Consumers have reported acceptable mouth feel, appearance, taste, and overall opinion, for bakery products with up to 25% lupin flour [10, 23], and up to 40% for chickpea flour [4, 6, 19, 24]. However, taste and appearance acceptability of pea and green pea flour was reported t*ο* be lower [6, 25, 26]. Thus, the effect of different pulse species is unclear.

In addition to ranking of preference, the use of vocabulary is crucial in order to describe differences of sensory profiles between products. Conventional descriptive profile methods are commonly used to characterize and quantify sensory similarities and differences between products. However, these methodologies are time-consuming, costly, and require training. Thus, Flash profile, which is a simpler descriptive method that offers a relative sensory positioning of samples, based on Free-Choice Profiling in combination with comparative evaluation [27] could be more appropriate to the industry. Flash profile involves ranking and discrimination by direct comparison of a simultaneously presented sample set and does not require consensual attributes [28]. It has been applied in many products such as jam, honey, cheese, and other dairy, in order to compare samples and/or panels of different cultures [27, 29–31].

Differences could be expected in the sensorial perception of crackers as a result of wheat replacement by pulse flours. It was hypothesized that changes in the sensorial perception could be driven by (a) the level of substitution and/or (b) species of pulses. Thus, a case study of comparing two pulse species at different levels of substitution was studied. The aim of this study was to investigate the effect of wheat substitution in crackers with varying concentrations of chickpea (40 to 80%) and lupin flours (10 to 30%), on physicochemical characteristics and sensory perception. Texture and color analysis of the samples were conducted along with Flash Profile sensory analysis in order to indicate possible associations between recipe alteration and product profiles with the intention to provide insight to strategies for developing products of wheat substitution by legumes. The comparison of two different panels intended to provide insight into how the perception of these products is affected by culture and differences in vocabulary.

## 2. Materials and Methods

### 2.1. Materials

Wheat flour (*triticum durum*) was made of “Lemnos” variety (Lemnos, Greece) and contained per 100 g on a dry basis: carbohydrate 70.0 g, protein 11.0 g, fat 1.4 g, fiber 4.0 g, and ash 1.0 g. Chickpea and lupin flour were purchased from commercial suppliers. Chickpea flour (*Cicer* arietinum) contained per 100 g on a dry basis: carbohydrate 44.5 g, protein 21.5 g, fat 6.0 g, and fiber 17.0 g. Lupin flour (*L. albus*) contained per 100 g on a dry basis: carbohydrate 11.0 g, protein 42.0 g, fat 14.0 g, and fiber 30.0 g. Levels of substitution of wheat were selected based on preliminary trials of baking (data not shown) so that the descriptive analysis provided data from a wide range of flour percentages.

### 2.2. Sample Preparation

All cracker samples were made based on the same formulation by substituting wheat with 40 to 80% chickpea and 10 to 30% lupin flour. Crackers made out of 100% wheat flour were used as control samples. The formulation of ingredients was as follows: flour 60.7%, water 24.3%, canola oil 12.1%, baking powder 1.1%, salt 0.6%, and sugar 1.2%. Baking powder was containing corn starch and as bulking agents disodium diphosphate and sodium hydrogen carbonate. The formulation of the samples was as follows: 100% wheat flour (WF), 40% chickpea flour (CF), 60% CF, 80% CF, 10% lupin flour (LF), 20% LF, and 30% LF. All ingredients were combined in a dough by mixing using KMC570 (Kenwood, United Kingdom) mixer machine for 8 minutes and allowed to rest for 30 minutes. After rest, the dough was sheeted (thickness 2 mm) using a manual dough molding machine (Hendi) and had been cut in dimensions 10 × 7.5. Nine punches were made in each sample. Cracker dimensions, including length, width, and thickness, were measured with a digital caliper and the spread ratio was calculated according to:

(1) $$\begin{array}{c} \text{Spread ratio}=\frac{\text{width}}{\text{thickness}} \end{array}$$

and presented in Table S2.

Samples were baked at 170° C for 15 minutes in an electric heating air oven (North, FK-60W). The samples were allowed to cool at room temperature for 30 minutes and were stored in polyethylene bags at 20°C.

Each formulation was prepared thrice on different days and the physicochemical analyses were conducted 20 hours after baking.

### 2.3. Sensory Analysis

#### 2.3.1. Panel

Greek and Indonesian untrained panels took part in the study comprising 24 judges (14 females and 10 males) and 22 judges (17 females and 5 males), respectively, after completing a consent form. The age of the judges was between 21 and 55 years old. The judges were food experts from the University of the Aegean and Indonesia International Institute for Life Sciences (i3L). Sensory evaluations of Greek and Indonesian panels were conducted in individual booths, at constant temperature (25°C) and lighting at the Laboratory of Consumer and Sensory Perception of Food & Drinks, University of Aegean, Lemnos, Greece, and i3L, respectively. Judges were informed that the samples were crackers and were asked to consider in-mouth flavor and texture.

#### 2.3.2. Flash Profile

Flash profile (FP) was conducted as described by Dairou and Sieffermann [27]. The analysis was composed of three sessions, with a briefing before each session. In Session 1, each judge created their own provisional list of attributes. Coded samples were presented simultaneously and judges were asked to list the sensory characteristics that best described their differences avoiding hedonic terms (e.g., like, dislike, and pleasant). During Session 2, all attributes were pooled into a single list and presented to the judges. They updated their personal lists by adding, excluding, or replacing attributes by comparison with the pooled list. Judges proceeded to rank the samples on a scale for each attribute individually using their own definitive attribute list. Session 3 was a repeat of the ranking. Each session lasted 20–30 minutes. Breaks were allowed and ties were permitted during ranking. Judges could evaluate and/or retaste the samples, in any order, as many times as they needed. Samples were presented in randomized order.

### 2.4. Texture Analysis

Hardness, fracturability, and total work of the crackers were measured using the Texture Analyzer (TA.XT. plus C, Stable Micro Systems, Surrey, UK) equipped with the Warner Bratzler blade (HDP/BS). Data were evaluated using the Texture Exponent Software (Version 6.1.18.0, Stable Micro Systems). The cracker was placed on the slotted blade insert and the blade moved downwards at a speed of 1 mm/sec and at a force load of 5 mm/sec, until the cracker fracture was achieved. Hardness was calculated as the maximum force required to break the sample. Fracturability (the distance at the point of break) and total energy (total area work) were also determined.

### 2.5. Color Analysis

The color of the samples was analyzed using a Lovibond LC100 Spectrocolorimeter. L∗ (0 = black, 100 = white), a∗ ([+] value = red, [-] value = green), b∗ ([+] value = yellow, [-] value = blue), h∗ (hue angle), and C∗ (chroma) values were recorded. Color analysis was performed by measuring six crackers from each batch at three different points. Browning index values were calculated as described by Wani and Kumar [32].

(2) $$\begin{array}{c} \text{Browning index}=\left(100\left[X-0.31\right]/0.17\right), \end{array}$$

where *X* = (a∗+1.75 L∗)/(5.645 L∗+a∗−3.012b∗).

### 2.6. Data Analysis

Physicochemical characteristics were measured in triplicate for each formulation and tested by one-way analysis of variance (ANOVA). The discrimination efficiency of the attributes for each assessor was tested by ANOVA on the rank data. Attributes that were found not to discriminate between the samples were excluded from a particular judge's list. Judges' repeatability between the two sessions was tested by Spearman's correlation test [33]. Only the attributes with reproducible ranking between the sessions were considered. Judges with poor discrimination ability and repeatability were excluded from the data set. Generalized Procrustes Analysis (GPA) was applied for the consensus configuration between judges' sensory maps. GPA calculates a consensus from data matrices of a sensory profiling experiment. In the case of Flash profile, a data matrix corresponds to each judge. The GPA plot demonstrates how similar or different the samples were to each other according to their schematic interpretation. Data were collated in Microsoft Excel and analyzed with ANOVA, Spearman's correlation test, and GPA, using XLSTAT as software (Addinsoft).

## 3. Results

### 3.1. Effect of Pulse Flours on the Color and Texture of Crackers

Chickpea and lupin flours led to significant differences in L∗ values (Table 1). As chickpea flour concentration increased to 40%, L∗ values were reduced from 71.03 to 61.60. However, increasing chickpea flour from 60 to 80% raised L∗ values to 66.78, without exceeding the control sample. A similar pattern was observed in the case of lupin flour. *Α*ddition of up to 30% lupin resulted in a reduction of L∗ values (up to 63.78) while 40% substitution of wheat resulted in an increase in L∗. However, it seems that up to 20% substitution of wheat by any flour did not lead to difference in L∗values (Table 1). Chickpea flour increased a∗ values, regardless of concentration. Lupin flour affected a∗ values only at concentrations above 20%. In contrast to lupin flour, the addition of chickpea flour did not affect b∗ values. Browning index (BI) was increased as a result of the substitution of wheat, at any concentration, with chickpea and lupin flour (Table 1). Regarding the texture of samples, the addition of lupin flour did not affect hardness, fracturability, or total work (Table 2). Chickpea flour did not affect fracturability; however, it decreased hardness when substitution was higher than 60%. Total work was affected at chickpea flour concentrations above 40%.

### 3.2. Sensory Analysis of Crackers with Flash Profile

**Figure 1:**
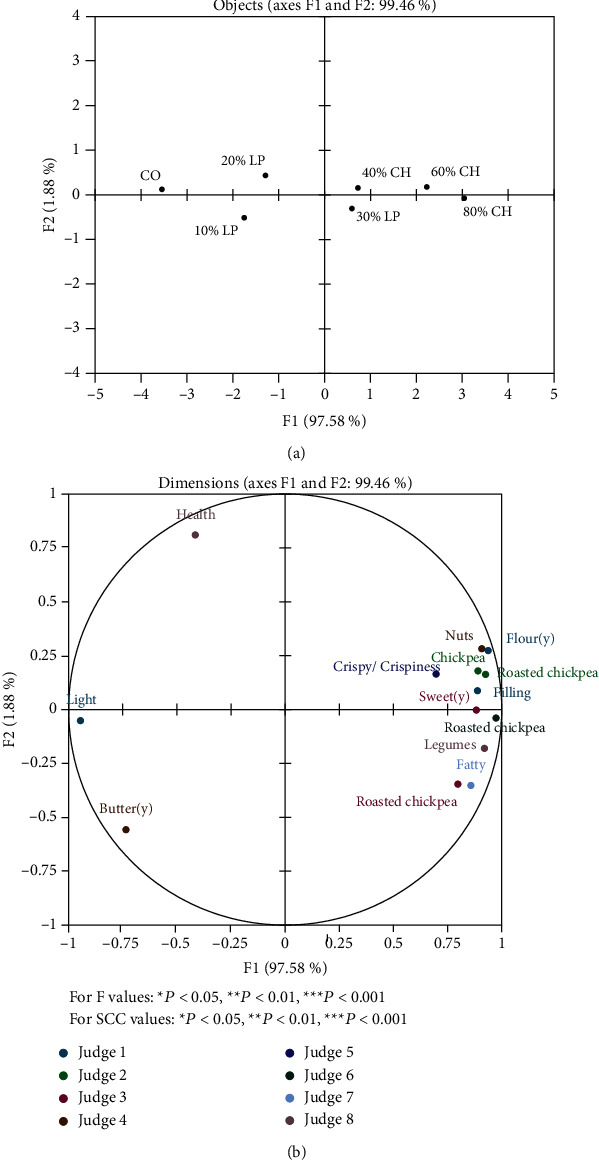
Generalized Procrustes Analysis (GPA) of Greek judge's evaluations of seven crackers samples via Flash profile analysis (a) and variable plot (b). Only attributes generated by judges with good repeatability and ability of discrimination were included. For *F* values: ^∗^*P* < 0.05, ^∗∗^*P* < 0.01, ^∗∗∗^*P* < 0.001. For SCC values: ∗*P* < 0.05, ^∗∗^*P* < 0.01, ^∗∗∗^*P* < 0.001.

**Figure 2:**
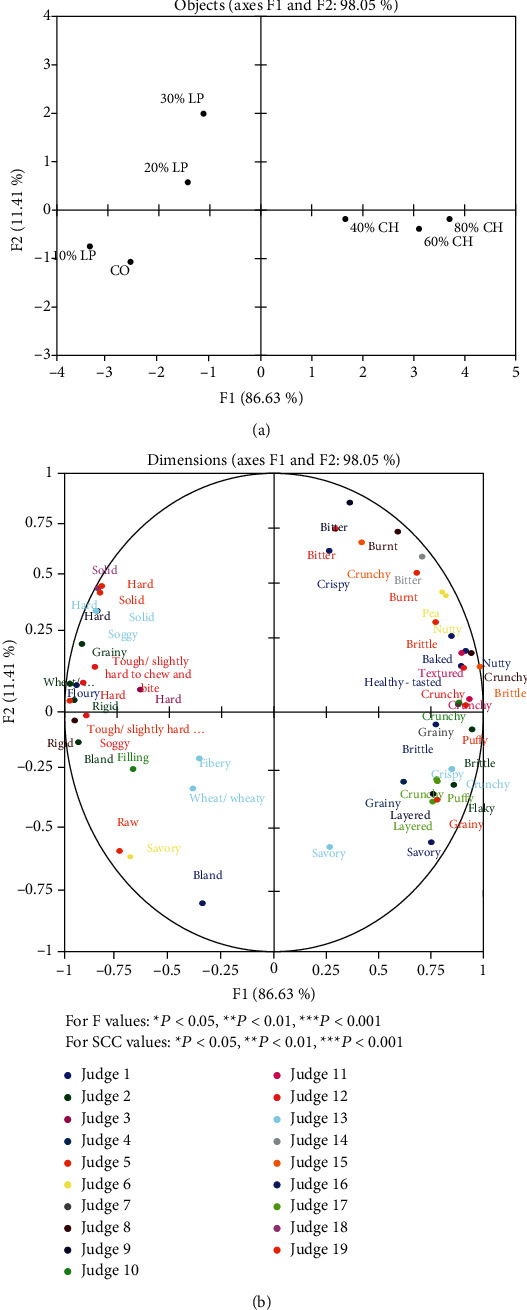
Generalized Procrustes Analysis (GPA) of Indonesian judge's evaluations of seven crackers samples via Flash profile analysis (a) and variable plot (b). Only attributes generated by judges with good repeatability and ability of discrimination were included. For *F* values: ^∗^*P* < 0.05, ^∗∗^*P* < 0.01, ^∗∗∗^*P* < 0.001. For SCC values: ^∗^*P* < 0.05, ^∗∗^*P* < 0.01, ^∗∗∗^*P* < 0.001.

During the first session, Greek judges generated 103 unique attributes, while Indonesian judges generated 49 attributes. The discrimination and repeatability of judges were evaluated via ANOVA and Spearman's rank correlation coefficient (SCC). Data from judges with poor repeatability and low discrimination were excluded from analysis. Statistical analysis showed that 19 Indonesian judges (15 females and 4 males) presented good discrimination of attributes and repeatability for each remaining attribute (Table 3). Eight Greek judges (6 females and 2 males) presented good discrimination of attributes and repeatability for each remaining attribute (Table 4.) Eight attributes were utilized by more than one Greek judge (Table 5). The GPA analysis of Greek panel revealed the relative positioning of the samples to be driven by the level of substitution with pulse flours. Factor F1 showed a high percentage of the total variance (97.58%) (Figure 1(a)). The control sample and crackers made with up to 20% substitution of wheat flour (LP10% and LP20%) were positioned on the left of F1 axis. In contrast, crackers made with 30% or more substitution of wheat were positioned on the opposite side of F1 regardless of legume species (LP30%, CH40, CH60, and CH80) (Figure 1(a)). According to GPA analysis of Indonesian panel, the plots defined by factor F1 explained a satisfactory percentage of the total variance (86.83%) (Figure 2(a)). Similarly, to Greek panel analysis, CO and LP10% and LP20% crackers were positioned on the left of F1 axis, while CH40, CH60, and CH80% were positioned on the left. On the contrary, LP30% crackers were positioned on the left side of F1 axis. Overall, crispiness seemed to be associated with particular samples, since it appeared in the right side of F1 (Figure 1(b)) for Greek panel. Similarly, generated attributes by Indonesian panel, like “Crispy,” “Crunchy,” and “Brittle” were associated with crackers made with high substitution of wheat (CH40, CH60, and CH80) (Figure 2(b)). Indonesian panel generated attributes like “Hard,” “Solid,” and “Rigid” in association with lower substitution of wheat; however, this has not emerged by Greek panel. As it would be expected, higher substitution of lupin and chickpea flours was associated with attributes relevant to pulses for both panels. Attributes such as “Legumes,” “Chickpea,” “Roasted chickpea,” and “Nuts” generated by Greek panel, while attributes “Nutty,” “Pea,” and “Grainy” generated by Indonesian panel in order to describe higher substitution of wheat flours. Attributes like “light” were associated with lower substitutions of lupin flour for Greek panel. Likewise, attribute “Bland” was associated with CO and LP10% samples for Indonesian panel. Most attributes were associated with the highest percentage of substitutions in both flours.

## 4. Discussion

According to Greek panel, GPA analysis showed differences between crackers to be driven by the level of wheat substitution. This suggests that judges were able to distinguish between different pulse flour concentrations while focusing less on different pulse flour varieties. On the contrary, GPA analysis indicated that Indonesian judges were able to segregate the different varieties of pulse flours. *Α*ttributes associated with texture, such as “Hard,” “Tough/slightly hard to chew and bite,” “Rigid,” and “Solid,” generated by Indonesian panel to describe crackers substituted with lupin flour and 100% wheat crackers (CO). This is in line with the instrumental analysis of texture in this study, as CO and lupin-based crackers had higher values in parameters of hardness and total work. It is important to note that hardness did not rose in response to substitutions with pulse flours in contrast to other studies [34–36] Crispiness is perceived when food is chewed between the molars and is usually expressed in terms of hardness and facturability [37]. For both panels, attribute “Crispy/crispiness” (Greek panel) or “Crispy” (Indonesian panel) was associated with crackers made by higher substitutions of wheat flour. As mentioned before, instrumental analysis of texture (both hardness and total work) presented significant differences between the samples. Therefore, it could be suggested that these differences were sensorially detectable by both panels. Bakery products made with chickpea and lupin flours achieved high rating scores regarding texture, like wheat crackers [4, 38], probably due to the higher concentration of protein compared to wheat-based recipes [39]. The addition of chickpea and lupin flour had an impact on the color of samples. Similar results were reported regarding L∗ values in bread substituted with up to 20% lupin flour [40]; however, up to 50% lupin flour in noodles did not affect L∗ values [41]. In this study, lupin flour led to increase in both a∗and b∗ values. Comparable results were reported in studies with instant noodles [41], pasta [23], and bread [40]. In agreement with previous reports, the addition of chickpea flour led to a reduction in L∗ while b∗ was not significantly influenced by it. The effect of chickpea flour on a∗ values was comparable in studies with similar varieties of pulses such as yellow pea [15]. Moreover, BI values showed a gradual increase as the substitution of chickpea and lupin flours rose. This may be attributed to Maillard browning reaction considering the higher percentage of protein in those flours [42]. Indeed, GPA analysis of Greek panel showed that attributes “Light” and “Butter(y)” were associated with the control sample and lower substitutions of wheat. GPA analysis of Indonesian panel indicated that attributes like “Burnt” and “Baked” were associated with higher substitutions of chickpea flour. On the contrary, as substitutions with above 30% lupin and 40% chickpea flour affected L∗ values, such attributes did not appear. Likewise, the attribute “Burnt” was used by judges; however, it was not included in GPA. It has been reported before that pulse flour leads to darker color of bakery products' surface [11]. Attributes related to legumes were generated by both panels despite they were unaware of the samples' composition. Moreover, the attributes “Well-chewed” and “Bitter” were generated by judges; however, these were not considered in GPA (Table 4). Comparable attributes such as “legume flavor” and “legume aroma” were used to describe biscuits made with lupin flour at a concentration ranging between 25 and 100% [10]. Similar or identical attributes (such as “crispy,” “chewy,” “tasteless,” “buttery appearance,” and “bitter taste”) were reported in the evaluation of bakery products with lupin flour [43]. More attributes emerged as chickpea and lupin concentration increased. This could be due to the flavor profile of these legumes. Studies have shown that chickpea, lupin seed, and lupin flours are characterized by a rich aroma profile [44–47].

## 5. Conclusions

Overall, there was a correlation between the results from the instrumental analysis of color and sensorial perception of crackers. It seems that there are minimum and maximum levels of wheat substitution that could be considered in substitution of wheat above which the sensorial profile is altered and pulse-related characteristics become detectable. Nevertheless, the way pulses affect the properties of crackers and their sensorial profile is not universal for all species nor all levels of substitution. In this respect, a lower concentration of lupin flour is required in order to affect sensory perception, while the use of chickpea flour seems to be detectable regardless of concentration. In this study, panels of two different cultures were compared to each other and differences in results had been observed. This highlights the need to consider culture effects.

## Figures and Tables

**Table 1:** Effects on color coordinates and Browning index of crackers by the addition of chickpea and lupine flour.

Samples	L∗	a∗	b∗	C∗	h∗	Browning index
CO	71.03^a^	6.74^c^	32.35^d^	33.80^e^	78.26^a^	66.20^e^
CH20%	64.18^bc^	11.08^a^	32.60^d^	34.51^de^	71.33^cde^	81.52^cd^
CH40%	61.60^c^	13.53^a^	33.25^d^	36.00^cd^	67.76^e^	91.06^abc^
CH60%	64.97^bc^	12.07^a^	32.66^d^	34.92^d^	69.70^de^	86.95^bcd^
CH80%	66.78^abc^	12.62^a^	33.90^d^	35.47^cd^	69.13^de^	82.71^cd^
LP10%	69.48^ab^	8.13^bc^	36.11^c^	37.04^c^	77.30^ab^	79.40^d^
LP20%	65.25^bc^	11.50^a^	37.82^bc^	39.58^b^	73.01^bcd^	95.71^ab^
LP30%	63.78^c^	11.54^a^	38.70^ab^	40.43^ab^	73.37^bcd^	102.15^a^
LP40%	66.25^abc^	10.96^a^	40.31^a^	41.80^a^	74.76^abc^	100.91^a^
Standard deviation	2.90	2.15	3.02	2.86	3.62	11.47

Means in a column followed by same letters (a, b, c, d, and e) are not significantly different (*P* > 0.05). Chickpea flour (CH), lupin flour (LP), 100% wheat flour-control (CO).

**Table 2:** Changes in texture parameters of crackers in response to replacement of wheat by chickpea and lupine flours.

Samples	Hardness (N)	Fracturability (mm)	Total work (joule)
CO	17.78^a^	2.37^a^	0.03^a^
CH20%	12.25^ab^	2.21^a^	0.02^abc^
CH40%	12.18^ab^	2.52^a^	0.01^bc^
CH60%	10.44^b^	3.13^a^	0.02^abc^
CH80%	9.76^b^	1.54^a^	0.01^c^
LP10%	14.22^ab^	2.83^a^	0.02^abc^
LP20%	12.26^ab^	2.23^a^	0.02^abc^
LP30%	15.185^ab^	2.40^a^	0.02^abc^
LP40%	16.18^ab^	2.70^a^	0.02^abc^
Standard deviation	2.67	0.45	0.01

Means in a column followed by same letters (a, b, and c) are not significantly different (*P* > 0.05). Chickpea flour (CH), lupin flour (LP), 100% wheat flour-control (CO).

**Table 3:** *F*-values (*P* < 0.05 for ANOVA) and SCC values (*P* < 0.05) for Spearman's correlation test on sensory attributes from each Indonesian judge in the Flash profile.

	*F*	SCC
Judge 1		
Renyah (crunchy)	0.802	
Gosong (burnt)	2.807	
Rasa tepung (floury)	13.912^∗∗∗^	0.860^∗^
Rasa mentega (buttery)	4.640^∗^	0.598
Keras (hard)	3.423	
Garing (crispy)	18.397^∗∗∗^	0.881^∗^
Rasa sehat (healthy-tasted)	124.833^∗∗∗^	0.981^∗∗^
Rasa kacang (nutty)	62.417^∗∗∗^	0.963^∗∗^
Gurih (savory)	6.093^∗∗^	0.679
Asin (salty)	4.773^∗^	0.607
Judge 2		
Kering (dry)	8.253^∗∗^	0.752
Kaku (rigid)	62.417^∗∗∗^	0.963^∗∗^
Rasa tepung/gandum (wheat/wheaty)	9.970^∗∗^	0.791^∗^
Hambar (bland)	34.833^∗∗∗^	0.935^∗∗^
Keras (hard)	2.574	
Berserpihan (flaky)	10.5^∗∗^	0.800^∗^
Pahit (bitter)	0.005	
Padat (solid)	4.507^∗^	0.589
Mudah patah (brittle)	14^∗∗∗^	0.846^∗^
Kasar (rough)	2.672	
Berpasir (grainy)	9.970^∗∗^	0.791^∗^
Judge 3		
Kering (dry)	3.967^∗^	0.546
Keras (hard)	21.742^∗∗∗^	0.898^∗∗^
Berpasir (grainy)	1.750	
Raca kacang (nutty)	2.481	
Rasa tepung (floury)	1.064	
Hambar (bland)	2.197	
Judge 4		
Beraroma (aromatic)	3.912^∗^	0.541
Kering (dry)	1.300	
Panggang (baked)	1.209	
Asin (salty)	0.428	
Gurih (savory	0.552	
Manis (sweet)	0.631	
Pahit (bitter)	0.442	
Berserpihan (flaky)	0.123	
Berpasir (grainy)	19.833^∗∗∗^	0.889^∗^
Kasar (rough)	2.818	
Rasa tepung/gandum (wheat/wheaty)	0.479	
Renyah (crunchy)	0.693	
Garing (crispy)	2.448	
Kaku (rigid)	8.253^∗∗^	0.753
Judge 5		
Mengembang (puffy)	15.167^∗∗∗^	0.857^∗^
Renyah (crunchy)	0.903	
Rasa kacang (nutty)	3.223	
Keras (hard)	2.287	
Mudah patah (brittle)	22.379^∗∗∗^	0.901^∗∗^
Rasa tepung/gandum (wheat/wheaty)	0.467	
Mentah (raw)	11.9^∗∗^	0.821^∗^
Judge 6		
Gurih (savory)	14.069^∗∗∗^	0.847^∗^
Rasa kacang (nutty)	27.093^∗∗∗^	0.917^∗∗^
Kacang polong (pea)	18.577^∗∗∗^	0.882^∗^
Berpasir (grainy)	2.608	
Kasar (rough)	2.608	
Keras (hard)	5.963^∗∗^	0.673
A lot (tough/slightly hard to chew and bite)	5.474^∗^	0.649
Tekstur tebal (thick texture)	0.308	
Judge 7		
Gosong (burnt)	2.381	
Manis (sweet)	4.278^∗^	0.571
Kacang polong (pea)	4.667^∗^	0.600
Keras (hard)	3.184	
Berpasir (grainy)	14.069^∗∗∗^	0.847^∗^
Judge 8		
Kering (dry)	3.889^∗^	0.539
Keras (hard)	5.574^∗^	0.654
Kaku (rigid)	46.900^∗∗∗^	0.952^∗∗^
Renyah (crunchy)	15.633^∗∗∗^	0.861^∗^
Rasa tepung/gandum (wheat/wheaty)	3.184	
Rasa kacang (nutty)	4.132^∗^	0.560
Pahit (bitter)	4.589^∗^	0.595
Manis (sweet)	1.167	
Gosong (burnt)	33.833^∗∗∗^	0.934^∗∗^
Lembut (tender)	5.704^∗∗^	0.661
Judge 9		
Keras (hard)	17.167^∗∗∗^	0.873^∗^
Berminyak (oily)	5.415^∗^	0.645
Pahit (bitter)	10.5^∗∗^	0.8^∗^
Berlayer (layered)	17.167^∗∗∗^	0.873^∗^
Berserpihan (flaky)	8.615^∗∗^	0.761
Judge 10		
Mengenyangkan (filling)	16.1^∗∗∗^	0.865^∗^
Rasa kacang (nutty)	9.006^∗∗^	0.771
Rasa tepung/gandum (wheat/wheaty)	6.099^∗∗^	0.679
Gurih (savory)	3.041	
Renyah (crunchy)	15.167^∗∗∗^	0.857^∗^
Judge 11		
Keras (hard)	1.803	
Padat (solid)	2.676	
Rasa tepung/gandum (wheat/wheaty)	1.803	
Hambar (bland)	1.803	
Renyah (crunchy)	15.167^∗∗∗^	0.857^∗^
Bertekstur (textured)	20.611^∗∗∗^	0.893^∗∗^
Garing (crispy)	1.346	
Judge 12		
A lot (tough/slightly hard to chew and bite)	18.756^∗∗∗^	0.883^∗^
Renyah (crunchy)	20.222^∗∗∗^	0.891^∗^
Keras (hard)	11.056^∗∗^	0.809^∗^
Melempem (soggy)	18.756^∗∗∗^	0.883^∗^
Hambar (bland)	2.917	
Gosong (burnt)	8.167^∗∗^	0.750
Pahit (bitter)	12.465^∗∗^	0.829^∗^
Judge 13		
Rasa lembut (creamy)	8.167^∗∗^	0.750
Renyah (crunchy)	64.167^∗∗∗^	0.964^∗∗^
Garing (crispy)	64.167^∗∗∗^	0.964^∗∗^
Padat (solid)	64.167^∗∗∗^	0.964^∗∗^
Asin (salty)	4.773^∗^	0.607
Manis (sweet)	8.167^∗∗^	0.750
Gurih (savory)	15.167^∗∗∗^	0.857^∗^
Rasa kacang (nutty)	2.100	
Melempem (soggy)	64.167^∗∗∗^	0.964^∗∗^
Keras (hard)	64.167^∗∗∗^	0.964^∗∗^
Rasa tepung/gandum (wheat/wheaty)	11.9^∗∗^	0.821^∗^
Berserat (fibery)	11.9^∗∗^	0.821^∗^
Pahit (bitter)	8.167^∗∗^	0.750^∗^
Judge 14		
Berpasir (grainy)	0.579	
Renyah (crunchy)	1.300	
Pahit (bitter)	20.611^∗∗∗^	0.893^∗∗^
Hambar (bland)	1.398	
Rasa tepung/gandum (wheat/wheaty)	0.334	
Judge 15		
Gosong (burnt)	6.854^∗∗^	0.709
Berpasir (grainy)	2.937	
Tepung haver (oat)	4.666^∗^	0.600
Rasa tepung/gandum (wheat/wheaty)	4.485^∗^	0.587
Renyah (crunchy)	27.611^∗∗∗^	0.919^∗∗^
Mudah patah (brittle)	18.756^∗∗∗^	0.883^∗^
Gurih (savory)	1.083	
Rasa sehat (healthy-tasted)	3.223	
Judge 16		
Hambar (bland)	9.993^∗∗^	0.791^∗^
Garing (crispy)	8.426^∗∗^	0.757
Panggang (baked)	31.500^∗∗∗^	0.929^∗∗^
Berserpihan (flaky)	2.100	
Mudah patah (brittle)	13.093^∗∗^	0.837^∗^
Mentah (raw)	7.000^∗∗^	0.714
Rasa mentega (buttery)	1.880	
Gurih (savory)	14.069^∗∗∗^	0.847^∗^
Rasa rempah (spice)	5.415^∗^	0.645
Berudara (airy)	4.180^∗^	0.564
Manis (sweet)	7^∗∗^	0.714
Keras (hard)	5.833^∗∗^	0.667
Renyah (crunchy)	3.223	
Melempem (soggy)	2.463	
Judge 17		
Mengembang (puffy)	20.611^∗∗∗^	0.893^∗∗^
Berlayer (layered)	11.9^∗∗^	0.821^∗^
Renyah (crunchy)	11.167^∗∗^	0.811^∗^
Asin (salty)	2.699	
Judge 18		
Padat (solid)	30.917^∗∗∗^	0.927^∗∗^
Gurih (savory)	3.454	
Melempem (soggy)	5.295^∗^	0.639
Renyah (crunchy)	1.333	
Mentah (raw)	0.555	
Asin (salty)	1.222	
Gosong (burnt)	2.263	
Judge 19		
Beraroma (aromatic)	1.803	
Asin (salty)	0.875	
Gosong (burnt)	15.167^∗∗∗^	0.857^∗^
Keras (hard)	50.633^∗∗∗^	0.955^∗∗^
Padat (solid)	31.500^∗∗∗^	0.929^∗∗^
Rasa tepung (floury)	2.917	
Alot (tough/slightly hard to chew and bite)	30.917^∗∗∗^	0.927^∗∗^
Berpasir (grainy)	17.167^∗∗∗^	0.878^∗^

For *F* values: ^∗^*P* < 0.05, ^∗∗^*P* < 0.01, ^∗∗∗^*P* < 0.001. For SCC values: ^∗^*P* < 0.05, ^∗∗^*P* < 0.01, ^∗∗∗^*P* < 0.001.

**Table 4:** *F*-values (*P* < 0.05 for ANOVA) and SCC values (*P* < 0.05) for Spearman's correlation test on sensory attributes from each Greek judge in the Flash profile.

Judge 1		
Flour(y)	64.166^∗∗∗^	0.964^∗∗^
Light	15.166^∗∗∗^	0.857^∗^
Crispy/crispiness	64.167^∗∗∗^	0.643
Neutral	15.166^∗∗∗^	0.857^∗^
Filling	31.500^∗∗∗^	0.929^∗∗^
Judge 2		
Chickpea	12.055^∗∗^	0.824^∗^
Flour(y)	0.284553	
Roasted chickpea	13.092^∗∗∗^	0.836^∗^
Cheese/cheesy	0.2625	
Burnt	0.6	
Judge 3		
Roasted chickpea	20.611^∗∗∗^	0.893^∗∗^
Fatty	8.167^∗∗^	0.750
Sweet(y)	64.167^∗∗∗^	0.964^∗∗^
Dietary	2.463	
Judge 4		
Granny	1.407	
Crispy/crispiness	0.482	
Butter(y)	15.633^∗∗∗^	0.861^∗^
Nuts	83.611^∗∗∗^	0.973^∗∗^
Flour(y)	0.683	
Judge 5		
Neutral	1.665	
Crispy/crispiness	27.093^∗∗∗^	0.917^∗∗^
Sweet(y)	0.749	
Olive oil	1.398	
Judge 6		
Crispy/crispiness	3.333	
Roasted chickpea	24.267^∗∗∗^	0.908^∗∗^
Pie/pastry sheet	5.587^∗∗^	0.655
Judge 7		
Fatty	11.055^∗∗^	0.809^∗^
Bitter	5.833^∗∗^	0.667
Zucchini	2.917	
Alcalic	2.676	
Legumes	7.333^∗∗^	0.727
Judge 8		
Lemnos	2.149	
Health	11.900^∗∗^	0.821^∗^
Legumes	10.056^∗∗^	0.795^∗^

For *F* values: ^∗^*P* < 0.05, ^∗∗^*P* < 0.01, ^∗∗∗^*P* < 0.001. For SCC values: ^∗^*P* < 0.05, ^∗∗^*P* < 0.01, ^∗∗∗^*P* < 0.001.

**Table 5:** Sensory attributes generated and used by more than one judge.

Attribute	Number of judges using the attribute
Crispy/crispiness	6
Flour(y)	4
Sweet(y)	4
Roasted chickpea	3
Dietary	2
Fatty	2
Legumes	2
Neutral	2

## Data Availability

The data (color and texture measurements, *F*-values, and Spearman's correlation coefficients (SCC)) used to support the findings of this study are included within the article. The raw data included in Table 5 are available from the corresponding author upon request.

## Additional Points

*Practical Applications.* Flash profile has allowed the observation of sensory characteristics correlated to the properties of the products and comparison of culture responses. Optimized combinations of type and amount of pulse flour could be utilized for the development of a desirable sensory profile for a product, which seems to be associated to physicochemical properties.

## Supplementary Materials

Supplementary Materials Table S1: Formulation of crackers used in Flash profile analysis and instrumental measurements. In Table S2 there are the dimensions of each cracker formulation. Figure S1.: Crackers made with increasing substitutions of lupin (a) and chickpea (b) flours. Chickpea flour (CH), Lupin flour (LP), 100% Wheat flour- Control (CO).
