# Supplementary material for: Effect of Wheat Replacement by Pulse Flours on the Texture, Color, and Sensorial Characteristics of Crackers: Flash Profile Analysis
Source: Int J Food Sci. 2022 Aug 18;2022:2354045. doi: 10.1155/2022/2354045 (PMC9410925; doi:10.1155/2022/2354045)

SUPPLEMENTARY

Effect of wheat replacement by pulse flours on the texture, color and sensorial characteristics of crackers: Flash profile analysis

Danai Ioanna Koukoumaki[1], Konstantinos Giannoutsos[1], Putu Virgina Partha Devanti[2] Panagiotis Karmiris[1], Sophia Bourni[1], Anastasia Monemvasioti[1], Vasiliki Psimouli[1], Dimitris Sarris[1], Konstantinos Gkatzionis[1] *

[1]Laboratory of Consumer and Sensory Perception of Food & Drinks, Department of Food Science and Nutrition, School of the Environment, University of the Aegean, Metropolite Ioakeim 2, GR 81400, Myrina, Lemnos, Greece

[2]Indonesia International Institute for Life Sciences, Jakarta 13210, Indonesia

**Corresponding author: Konstantinos Gkatzionis: kgkatzionis@aegean.gr*

Table S1. Formulation of crackers used in Flash profile analysis and instrumental measurements.

Samples	Wheat flour (g)	Chickpea flour (g)	Lupin flour (g)	Canola oil (g)	Sugar (g)	Salt (g)	Baking powder (g)	Water (g)
Control	200	-	-	40	4	2	3.6	80
10% LF	180	-	20	40	4	2	3.6	80
20% LF	160	-	40	40	4	2	3.6	80
30% LF	140	-	60	40	4	2	3.6	80
40% CH	120	80	-	40	4	2	3.6	80
60% CH	60	120	-	40	4	2	3.6	80
80% CH	40	160	-	40	4	2	3.6	80

Chickpea flour (CH), Lupin flour (LP), 100% Wheat flour- Control (CO)

Table S2. Dimensions of each cracker formulation

**Samples**	**Thickness- T (mm)**	**Width- W (mm)**	**Length – L (mm)**	**Spread ratio (W/T)**
**CO**	32.349 ^{a}	70.133 ^{b}	103.727 ^{abc}	2.173 ^{c}
**CH20%**	28.367 ^{ab}	70.030 ^{b}	104.180 ^{ab}	2.526 ^{abc}
**CH40%**	26.100 ^{b}	70.250 ^{b}	104.110 ^{ab}	2.693 ^{ab}
**CH60%**	24.550 ^{b}	70.095 ^{b}	103.985 ^{ab}	2.862 ^{a}
**CH80%**	25.133 ^{b}	72.150 ^{a}	105.077 ^{a}	2.873 ^{a}
**LP10%**	31.533 ^{a}	69.263 ^{b}	102.533 ^{bcd}	2.199 ^{c}
**LP20%**	28.672 ^{ab}	69.960 ^{b}	103.270 ^{bc}	2.441 ^{bc}
**LP30%**	31.143 ^{a}	69.949 ^{b}	102.000 ^{cd}	2.268 ^{c}
**LP40%**	29.056 ^{ab}	70.396 ^{b}	101.421 ^{d}	2.428 ^{bc}
**Standard deviation**	3.35	0.96	1.42	0.30

Means in a column followed by same letters (a,b,c) are not significantly different ($P > 0.05$).

Chickpea flour (CH), Lupin flour (LP), 100% Wheat flour- Control (CO)

Figure S1. Crackers made with increasing substitutions of lupin (a) and chickpea (b) flours.
Chickpea flour (CH), Lupin flour (LP), 100% Wheat flour- Control (CO)

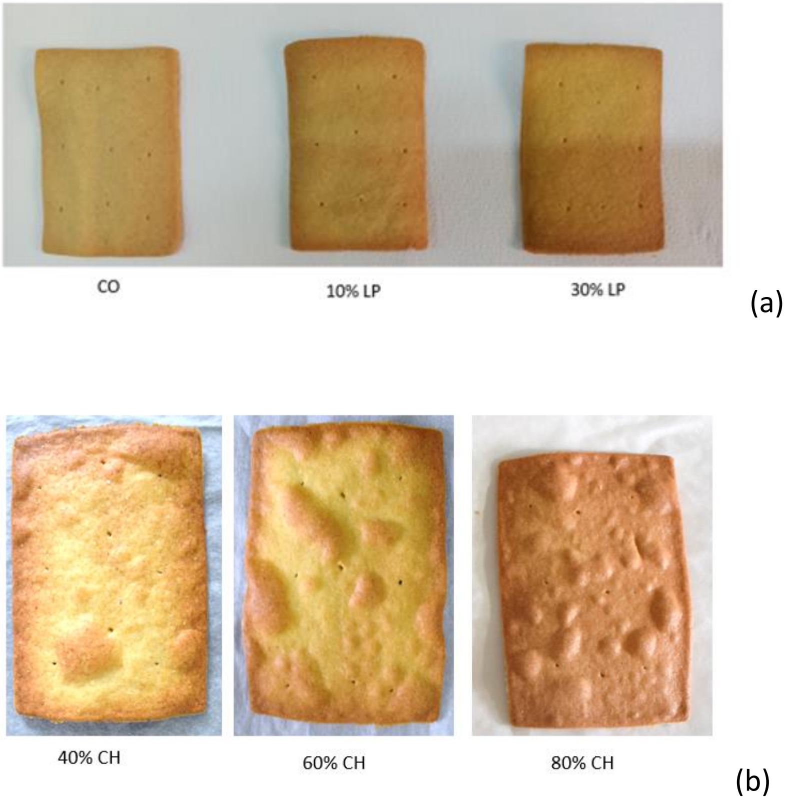

Supplement: Supplementary Materials — Table S1: Formulation of crackers used in Flash profile analysis and instrumental measurements. In Table S2 there are the dimensions of each cracker formulation. Figure S1.: Crackers made with increasing substitutions of lupin (a) and chickpea (b) flours. Chickpea flour (CH), Lupin flour (LP), 100% Wheat flour- Control (CO). [file 2354045.f1.zip › Supplementary.pdf]
