# Supplementary material for: Effect of Wheat Replacement by Pulse Flours on the Texture, Color, and Sensorial Characteristics of Crackers: Flash Profile Analysis
Source: Int J Food Sci. 2022 Aug 18;2022:2354045. doi: 10.1155/2022/2354045 (PMC9410925; doi:10.1155/2022/2354045)
Supplement: Supplementary Materials — Table S1: Formulation of crackers used in Flash profile analysis and instrumental measurements. In Table S2 there are the dimensions of each cracker formulation. Figure S1.: Crackers made with increasing substitutions of lupin (a) and chickpea (b) flours. Chickpea flour (CH), Lupin flour (LP), 100% Wheat flour- Control (CO). [file 2354045.f1.zip › v10.1 Supplementary (1).docx]

**SUPPLEMENTARY**

Effect of wheat replacement by pulse flours on the texture, color and sensorial characteristics of crackers: Flash profile analysis

Danai Ioanna Koukoumaki^1^, Konstantinos Giannoutsos^1^, Putu Virgina Partha Devanti^2^ Panagiotis Karmiris^1^, Sophia Bourni^1^, Anastasia Monemvasioti^1^, Vasiliki Psimouli^1^, Dimitris Sarris^1^, Konstantinos Gkatzionis^1 *^

*^1^Laboratory of Consumer and Sensory Perception of Food & Drinks, Department of Food Science and Nutrition, School of the Environment, University of the Aegean, Metropolite Ioakeim 2, GR 81400, Myrina, Lemnos, Greece*

*^2^Indonesia International Institute for Life Sciences, Jakarta 13210, Indonesia*

**Corresponding author: Konstantinos Gkatzionis: kgkatzionis@aegean.gr*

Table S1. Formulation of crackers used in Flash profile analysis and instrumental measurements.

| **Samples** | **Wheat flour (g)** | **Chickpea flour (g)** | **Lupin flour (g)** | **Canola oil (g)** | **Sugar (g)** | **Salt (g)** | **Baking powder (g)** | **Water (g)** |
| --- | --- | --- | --- | --- | --- | --- | --- | --- |
| Control | 200 | - | - | 40 | 4 | 2 | 3.6 | 80 |
| 10% LF | 180 | - | 20 | 40 | 4 | 2 | 3.6 | 80 |
| 20% LF | 160 | - | 40 | 40 | 4 | 2 | 3.6 | 80 |
| 30% LF | 140 | - | 60 | 40 | 4 | 2 | 3.6 | 80 |
| 40% CH | 120 | 80 | - | 40 | 4 | 2 | 3.6 | 80 |
| 60% CH | 60 | 120 | - | 40 | 4 | 2 | 3.6 | 80 |
| 80% CH | 40 | 160 | - | 40 | 4 | 2 | 3.6 | 80 |

Chickpea flour (CH), Lupin flour (LP), 100% Wheat flour- Control (CO)

Table S2. Dimensions of each cracker formulation

| **Samples** | **Thickness- T (mm)** | **Width- W (mm)** | **Length – L (mm)** | **Spread ratio (W/T)** |
| --- | --- | --- | --- | --- |
| **CO** | 32.349 ^a^ | 70.133 ^b^ | 103.727 ^abc^ | 2.173 ^c^ |
| **CH20%** | 28.367 ^ab^ | 70.030 ^b^ | 104.180 ^ab^ | 2.526 ^abc^ |
| **CH40%** | 26.100 ^b^ | 70.250 ^b^ | 104.110 ^ab^ | 2.693 ^ab^ |
| **CH60%** | 24.550 ^b^ | 70.095 ^b^ | 103.985 ^ab^ | 2.862 ^a^ |
| **CH80%** | 25.133 ^b^ | 72.150 ^a^ | 105.077 ^a^ | 2.873 ^a^ |
| **LP10%** | 31.533 ^a^ | 69.263 ^b^ | 102.533 ^bcd^ | 2.199 ^c^ |
| **LP20%** | 28.672 ^ab^ | 69.960 ^b^ | 103.270 ^bc^ | 2.441 ^bc^ |
| **LP30%** | 31.143 ^a^ | 69.949 ^b^ | 102.000 ^cd^ | 2.268 ^c^ |
| **LP40%** | 29.056 ^ab^ | 70.396 ^b^ | 101.421 ^d^ | 2.428 ^bc^ |
| **Standard deviation** | 3.35 | 0.96 | 1.42 | 0.30 |

Means in a column followed by same letters (a,b,c) are not significantly different (P > 0.05).

Chickpea flour (CH), Lupin flour (LP), 100% Wheat flour- Control (CO)

Figure S1. Crackers made with increasing substitutions of lupin (a) and chickpea (b) flours. Chickpea flour (CH), Lupin flour (LP), 100% Wheat flour- Control (CO)


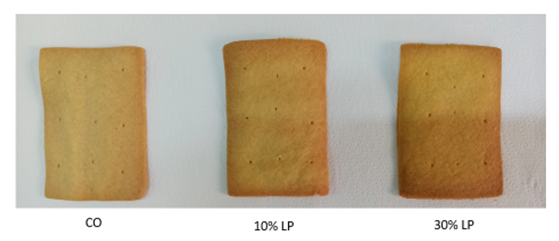
 (a)


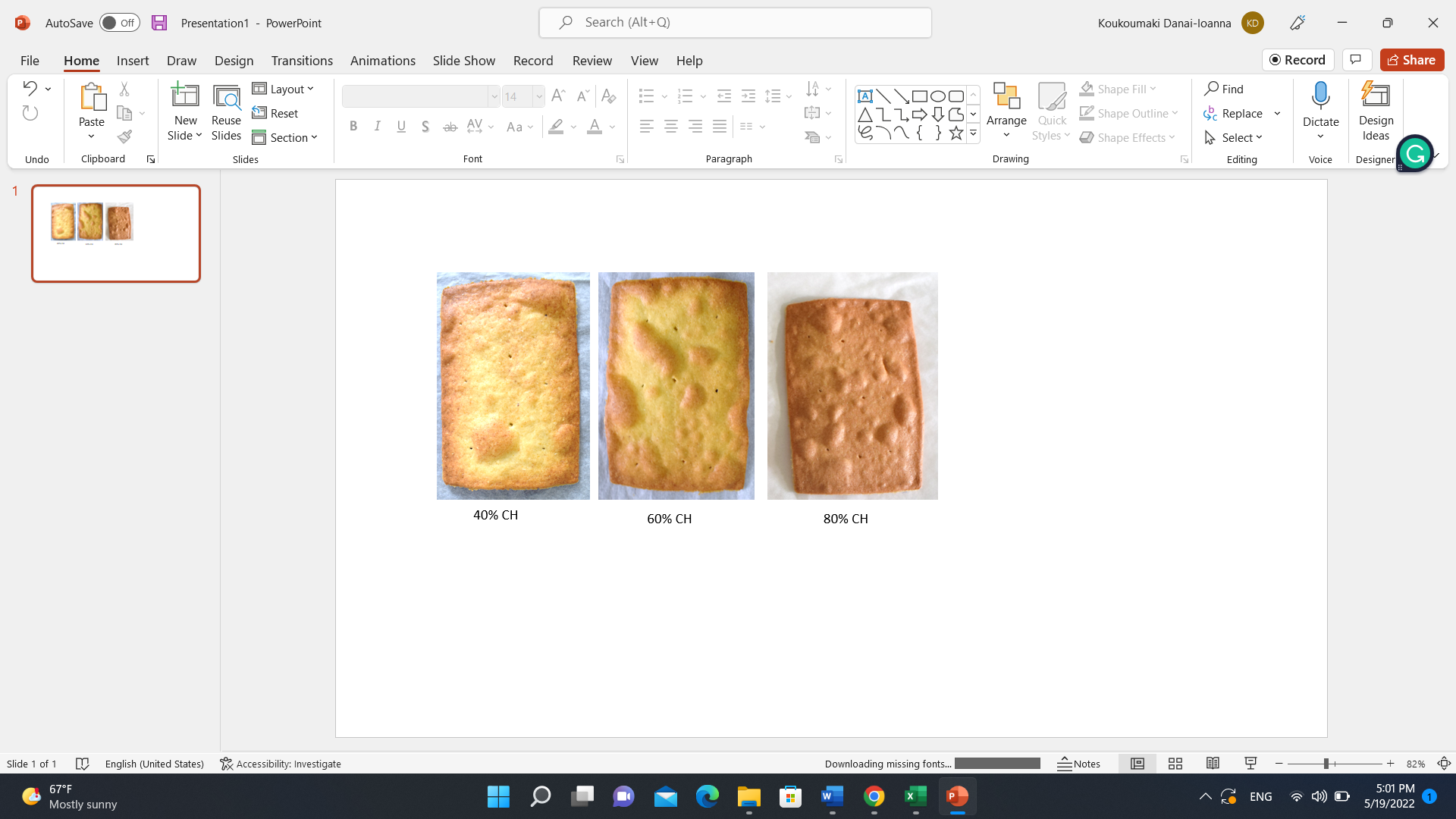
(b)
